# Improved Detection of Cytokines Produced by Invariant NKT Cells

**DOI:** 10.1038/s41598-017-16832-1

**Published:** 2017-11-30

**Authors:** Duygu Sag, Müge Özkan, Mitchell Kronenberg, Gerhard Wingender

**Affiliations:** 1 Izmir Biomedicine and Genome Center (IBG), 35340 Balcova, Izmir Turkey; 2 Department of Medical Biology, Faculty of Medicine, Dokuz Eylul University, 35340 Balcova, Izmir Turkey; 3 Izmir International Biomedicine and Genome Institute (IBG-Izmir), Dokuz Eylul University, 35340 Balcova, Izmir Turkey; 4 La Jolla Institute for Allergy and Immunology (LJI), 9420 Athena Circle, La Jolla, CA 92037 USA; 5 Division of Biological Sciences, University of California San Diego, La Jolla, CA 92037 USA

## Abstract

Invariant Natural killer T (*i*NKT) cells rapidly produce copious amounts of multiple cytokines after *in vivo* activation, allowing for the direct detection of a number of cytokines directly *ex vivo*. However, for some cytokines this approach is suboptimal. Here, we report technical variations that allow the improved detection of IL-4, IL-10, IL-13 and IL-17A *ex vivo*. Furthermore, we describe an alternative approach for stimulation of *i*NKT cells *in vitro* that allows a significantly improved detection of cytokines produced by *i*NKT cells. Together, these protocols allow the detection of *i*NKT cell cytokines *ex vivo* and *in vitro* with increased sensitivity.

## Introduction

Invariant natural killer T (*i*NKT) cells are innate-like T cells that share features with natural killer (NK) cells and memory T lymphocytes. Antigens for *i*NKT cells are largely glycolipids, like the model antigen α-galactosylceramide (αGalCer), which are presented by CD1d, a non-polymorphic MHC class I homolog. Following antigenic activation, subsets of *i*NKT cells rapidly produce copious amounts of cytokines, including Th1, Th2 and Th17 cytokines as well as IL-10^1–6^. However, the detection of some of these cytokines via intracellular cytokine staining (ICCS) is often poor, which is especially the case after *in vitro* activation of primary *i*NKT cells. This has led to inconsistencies in the published data for the production of several *i*NKT cell cytokines and hampers the use of *in vitro* assays to study the functions of primary *i*NKT cells. We present here several enhancements to purification and staining protocols that allow improved detection of cytokines, including GM-CSF, IFNγ, IL-2, IL-4, IL-10, IL-13, IL-17A and TNF, produced by primary *i*NKT cells, *ex vivo* or *in vitro*.

## Results

### Influence of the fixation method on cytokine detection

**Figure 1:**
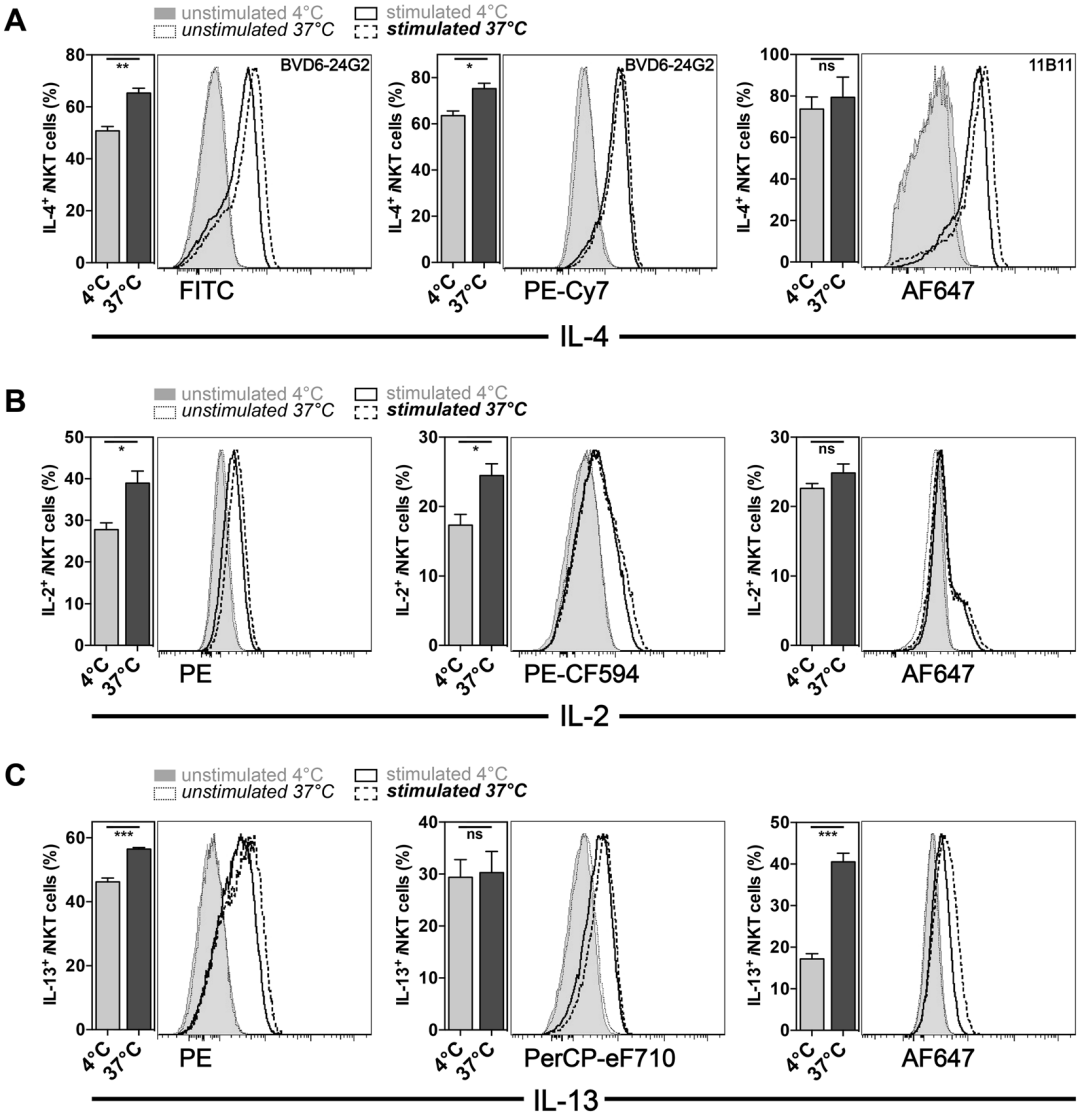
Fixation method influenced the detection in *i*NKT cells of several cytokines. **(A**–**C)** C57BL/6 mice were either mock treated or injected i.v. with 1μg αGalCer and 90 min later the expression of the cytokines IL-4 (clone BVD6-24G2 or 11B11, as indicated in the histogram) (**A**), IL-2 (JES6-5H4) (**B**) and IL-13 (13A) (**C**) by splenic *i*NKT cells was analyzed by intracellular cytokine staining (ICCS). Cells were fixed with Cytofix/Cytoperm for 10 min at either 4 °C or 37 °C as indicated. A summary graph (left) and representative data from gated *i*NKT cells (right) are in adjacent panels. The fluorochromes conjugated to the antibodies utilized are indicated below the histograms. ns = not statistically significant. Representative data from one of at least three independent experiments are shown.

Following activation with αGalCer *in vivo* the majority of *i*NKT cells produced IL-4, which can be detected directly *ex vivo*, meaning without the need for TCR cross-linking or pharmacologic activators, and without a requirement for culture in the presence of blockers of protein transport through the Golgi apparatus (Fig. 1A). However, the intensity of the staining tended to be low (Fig. 1A and data not shown) and this can at times make the discrimination of positive events difficult. Therefore, we tested several alternatives for staining and fixation to improve the intracellular staining for IL-4, combined with the use of different fluorophores. We found that fixation of the cells with Cytofix/Cytoperm for 10 minutes at 37 °C, instead of the recommended 4 °C, also significantly increased the staining intensity for most IL-4 conjugates, without negatively affecting surface staining (Fig. 1A and data not shown). This was seen with both of the αIL-4-antibody clones, 11B11 and BVD6-24G2, that were tested (Fig. 1A). The increased staining intensity allowed significantly more *i*NKT cells to be detected as IL-4^+^ in the case of FITC- and PE-Cy7-conjugated antibodies, but not in the case of AF647- and PE-CF594-conjugated antibodies (Fig. 1A and Supplementary Figure 1A). A similar variability in the percent of the activated *i*NKT cells classified as cytokine positive also was noted for IL-2 and IL-13 staining. This depended on the antibody conjugates tested, and for some of the conjugates, fixation at 37 °C led to an increased staining intensity (Fig. 1B). In contrast, no difference in the staining intensity of other cytokine tested, namely GM-CSF, IFNγ, IL-10, IL-17A and TNF, was observed (Fig. 1B, Supplementary Figure 1B, and data not shown). Importantly, changing the temperature of the fixation step did not negatively affecting the surface staining of any of the tested markers (Supplementary Figure 2). Therefore, fixation of activated *i*NKT cells at 37 °C instead of 4 °C is preferable for IL-2, IL-4 and IL-13 detection.

### *i*NKT cell IL-17A requires *in vitro* cytokine accumulation

**Figure 2:**
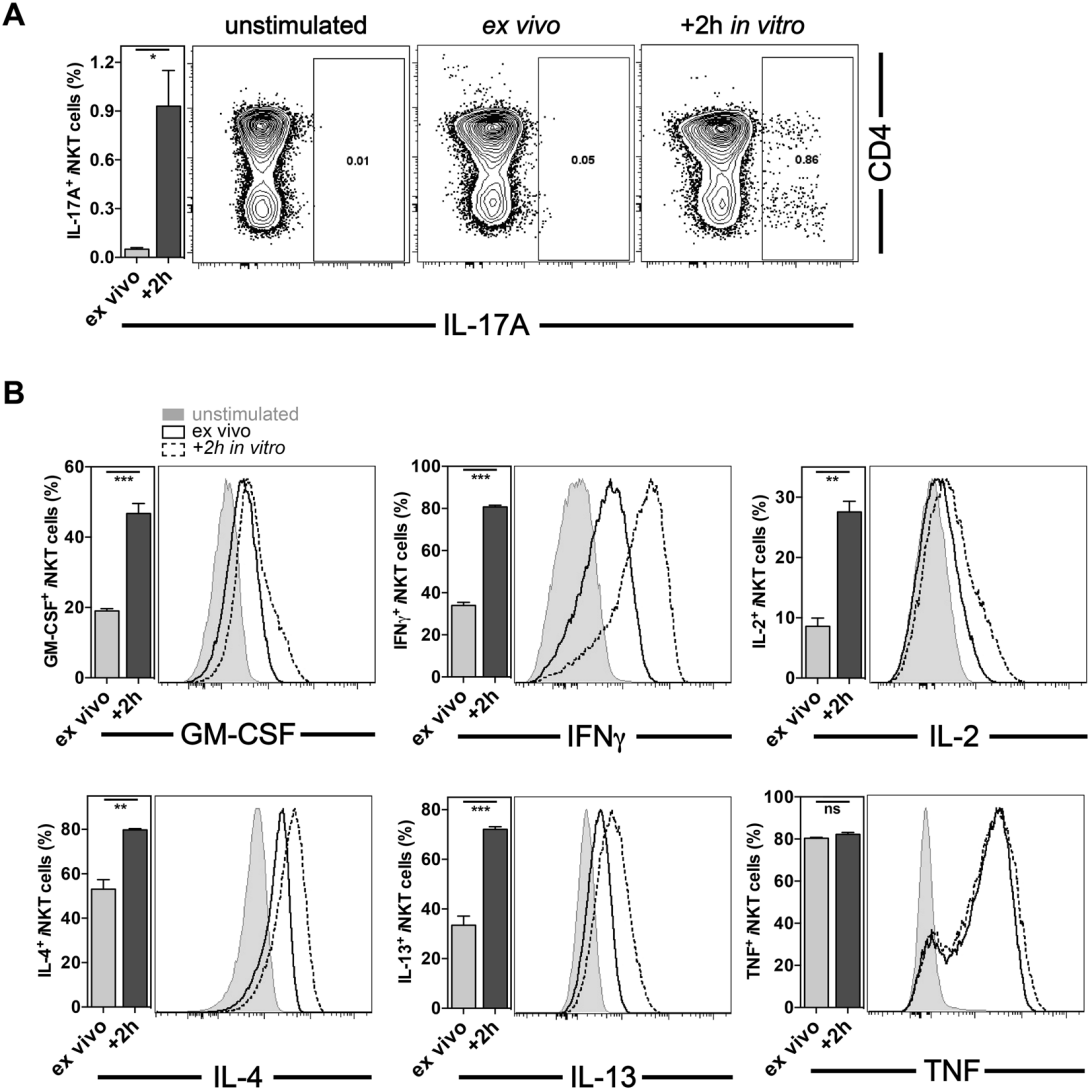
Effective detection of *i*NKT cell IL-17A *ex vivo* required cytokine accumulation *in vitro*. C57BL/6 mice were either mock treated or injected i.v. with 1μg αGalCer and 90 min later expression of the indicated cytokines by splenic *i*NKT cells was analyzed by ICCS. Cells were either stained directly *ex vivo* (*ex vivo*) or after a 2 h *in vitro* incubation at 37 °C in the presence of the Golgi-transport inhibitors Brefeldin A and monensin (+2 h). **(A)** Intracellular IL-17A produced by gated *i*NKT cells is depicted against CD4 for representative data, and as a summary graph (left panels). (**B**) Production of indicated cytokines by *i*NKT cells is depicted as a summary graph (left panels) and representative data (right panels). ns = not statistically significant. Representative data from one of at least three independent experiments are shown.

**Figure 3:**
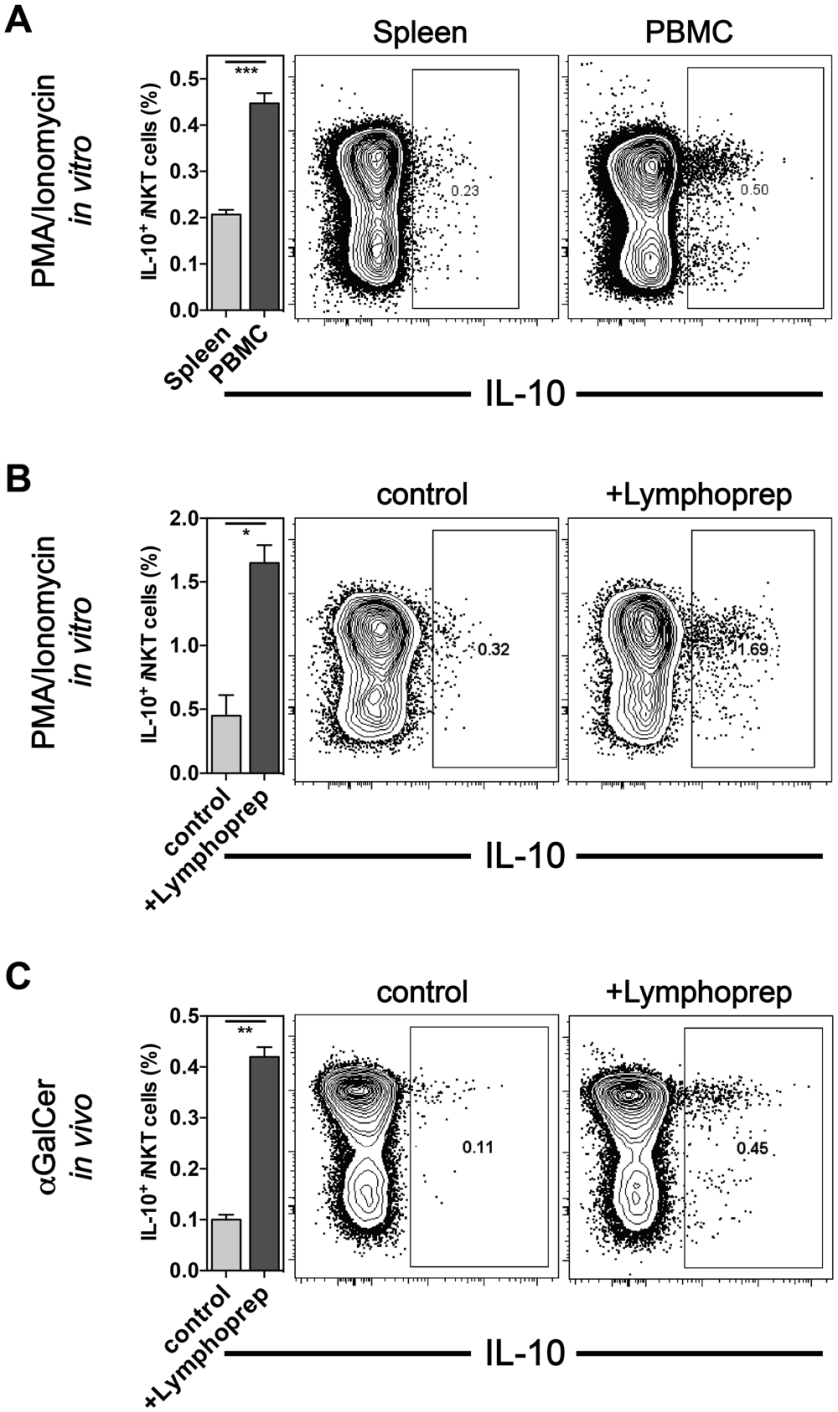
Effective detection of *i*NKT cell IL-10 required the removal of dead cells. (**A**) C57BL/6 splenocytes and PBMCs were stimulated *in vitro* for 4 h with PMA and ionomycin and the percentage of IL-10^+^ *i*NKT cells was measured by ICCS. (**B**) C57BL/6 splenocytes were either left untreated (control) or purified via a density-gradient (+Lymphoprep) and stimulated *in vitro* for 4 h with PMA and ionomycin in the presence of the Golgi-transport inhibitors Brefeldin A and monensin. The percentage of IL-10^+^ *i*NKT cells was then measured by ICCS. (**C**) C57BL/6 animals were injected i.v. with 1μg αGalCer and 90 min later splenocytes were purified via a density-gradient and incubation for 2 h *in vitro* in the presence of the Golgi-transport inhibitors Brefeldin A and monensin. The percentage of IL-10^+^ *i*NKT cells was then measured by ICCS. A summary graph (left panel) and representative data (right panel) are shown, respectively. The means of activation is also given on the left of the panels. Representative data from one of at least three independent experiments are shown.

In recent years, functional subsets of *i*NKT cells have been defined^7,8^. The definition of *i*NKT cell subsets is largely based on their expression of transcription factors and their function, especially significant biases in cytokine production of the respective *i*NKT cell types. NKT1^9^, NKT2^9,10^ and NKT17^11–14^ cells are defined as the *i*NKT cell subsets biased towards T_h_1, T_h_2 or T_h_17 cytokines, respectively. The underlying gene programs are imprinted during thymic development^15^. NKT10 cells were characterized by IL-10 production^16–20^. NKT_FH_^21–23^ and FoxP3^+^ *i*NKT^24,25^ cells were defined based on their similarities with T_FH_ and FoxP3^+^ T cells, respectively. However, the detection of IL-10 and IL-17A production by activated *i*NKT cells of the appropriate functional subtype is particularly poor when the cells were analyzed directly *ex vivo* (Figs 2A, 3A and data not shown). For the detection of cytokines produced by conventional, MHC class II-reactive T cells, an *in vitro* incubation of the cells after purification in the presence of Golgi-transport inhibitors is routinely used to improve cytokine detection^26^. We adopted this method for the detection of IL-17A production by *i*NKT cells. Mice were injection i.v. with αGalCer and 90 min later splenocytes were obtained and cultured for 2 h in the presence of Golgi-transport inhibitors. As shown in Fig. 2A, the IL-17A-producing subset is relatively infrequent in the spleen, but importantly, the *in vitro* accumulation of biosynthesized IL-17A was required for the effective detection of IL-17A^+^ *i*NKT cells. For several other cytokines tested, namely GM-CSF, IFNγ, IL-2, IL-4 and IL-13, a 2 h *in vitro* incubation resulted in a marked increase in the percentage of cytokine-positive *i*NKT cells (Fig. 2B). Also for IL-10 an improvement in cytokine staining was observed, which, however, did not reach statistical significance in all experiments (data not shown). In contrast, no difference was evident for TNF after a 2 h *in vitro* incubation (Fig. 2B). Furthermore, extending the *in vitro* incubation beyond 2 h did not improve cytokine detection further (data not shown). Therefore, *in vitro* culture of *in vivo* stimulated *i*NKT cells for 2 h in the presence of Golgi-transport inhibitors is required for efficient IL-17A detection and clearly improves the detection of most other *i*NKT cell cytokines.

### Effective detection of *i*NKT cell IL-10 requires dead cell removal

IL-10-producing *i*NKT cells are a relatively small subset in the spleen, but they are enriched in adipose tissue and increased long term after strong or repeated antigenic stimulation^19^. We noticed previously that the maximal number of IL-10^+^ *i*NKT cells could be detected after stimulation *in vitro* with PMA and ionomycin^19^. However, when we compared the IL-10 staining after PMA/ionomycin stimulation *in vitro* in *i*NKT cells from splenocytes and peripheral blood mononuclear cells (PBMCs) we noted a clearly stronger IL-10 staining in *i*NKT cells derived from PBMCs compared to splenocytes (Fig. 3A). As we did not expect such a difference in the *i*NKT cells present in PBMCs compared to the spleen, we tested if the different purification methods employed could account for the observed difference. Whereas splenocytes were utilized directly after the single cell suspension was obtained, PBMCs were first purified via a density-gradient to remove red blood cells and dead cells. Therefore, we compared the IL-10 staining after PMA/ionomycin stimulation *in vitro* of splenic *i*NKT cells that were used either directly *ex vivo* or after purification via a density-gradient. As shown in Fig. 3B the IL-10 staining in *i*NKT cells significantly improved, with regard to the percentage of IL-10^+^ cells detected as well as to the intensity of the staining, when dead cells were removed from the splenocytes prior of the stimulation. Given these results, we tested if the removal of dead cells would also allow an improved detection of IL-10^+^ *i*NKT cells *ex vivo* after αGalCer injection. Mice were injected i.v. with αGalCer and 90 min later splenocytes were obtained and analyzed either directly *ex vivo* or after purification via a density-gradient. To allow for accumulation of IL-10 in the *i*NKT cells, the splenocytes were cultured for 2 h *in vitro* in the presence of Golgi-transport inhibitors. Again, the purification via a density-gradient allowed an improved detection of IL-10^+^ cell *i*NKT cells (Fig. 3C). Therefore, for the optimal detection of IL-10, the initial removal of dead cells via a density-gradient and incubation *in vitro* in the presence of Golgi-transport inhibitors were required.

### Dead cell removal allows for improved detection of multiple cytokines

**Figure 4:**
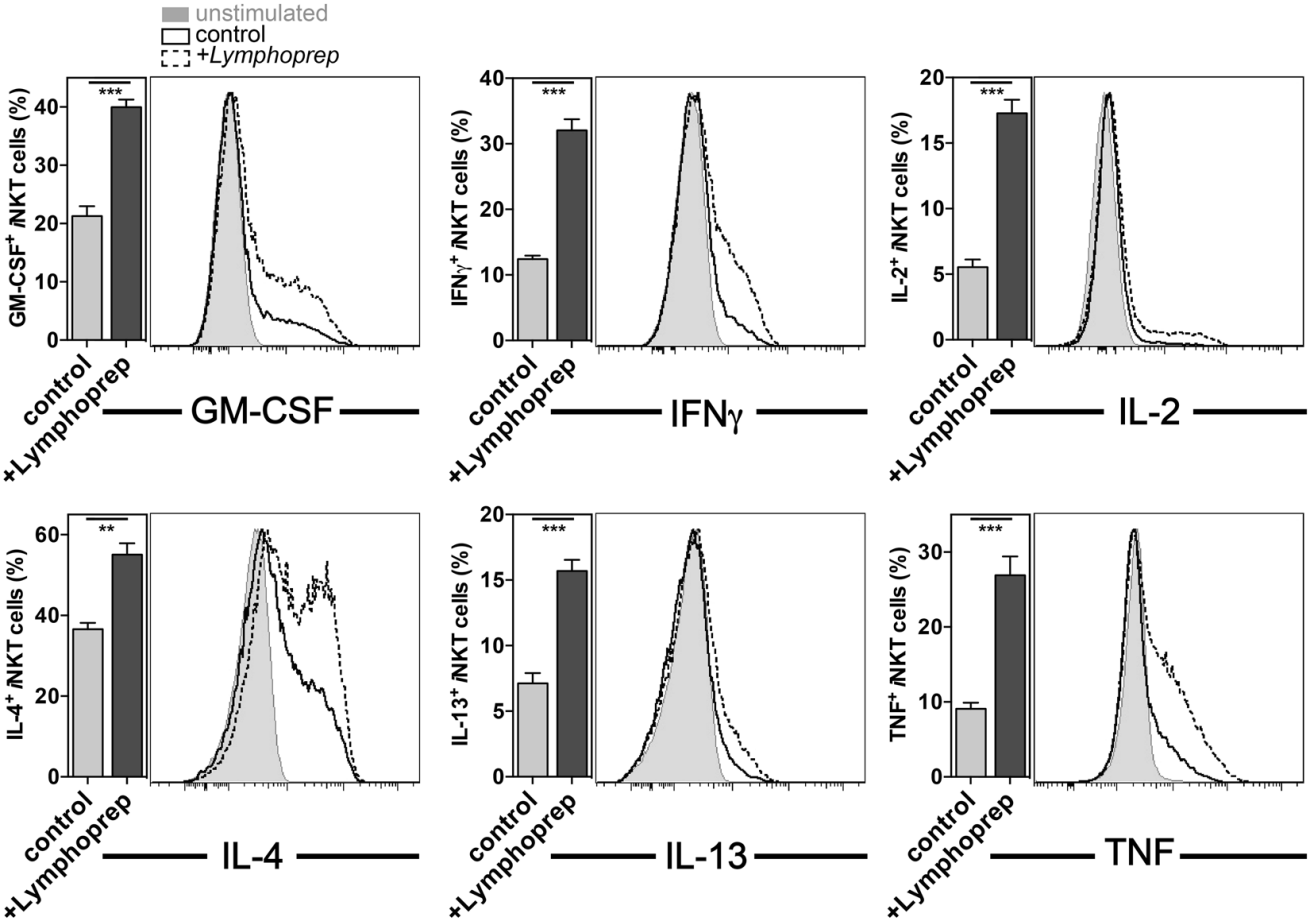
Removal of dead cells allowed for an improved detection of *i*NKT cell cytokines *in vitro*. C57BL/6 splenocytes were either left untreated (control) or purified via a density-gradient (+Lymphoprep) and stimulated *in vitro* for 5 h with 100ng/ml αGalCer in the presence of the Golgi-transport inhibitors Brefeldin A and monensin. The expression of the indicated cytokines by splenic *i*NKT cells was analyzed by ICCS. A summary graph (left panels) and representative data (right panels) are shown. Representative data from one of at least three independent experiments are shown.

Whereas the large majority of *i*NKT cells produce cytokines following activation with αGalCer *in vivo*, on a per cell basis their response after *in vitro* stimulation with αGalCer is weaker (Fig. 4 and data not shown). Given the clear improvement of the IL-10 staining by the elimination of dead cells, we tested whether a similar approach would improve cytokine detection by *i*NKT cells following *in vitro* stimulation with αGalCer. C57BL/6 splenocytes were either left untreated or purified by a density-gradient before the cells were incubated *in vitro* for 5 h in the presence of αGalCer and Golgi-transport inhibitors. As shown in Fig. 4, although the optimal *in vitro* stimulated responses did not reach the intensities observed when cells were analyzed *ex vivo*, significantly more *i*NKT cells from the gradient-purified splenocyte population scored positive for cytokine production after αGalCer stimulation. Additionally, the intensity of the cytokine staining obtained tended to be higher in *i*NKT cells from purified splenocytes (Fig. 4). The purification of splenocytes by a density-gradient either after αGalCer *in vivo* stimulation followed by a 2 h *in vitro* culture (Supplementary Figure 3A) or before *in vitro* stimulation with PMA and ionomycin (Supplementary Figure 3B) also allowed for increased detection of cytokine-positive *i*NKT cells. This increase in cytokine-positive *i*NKT cells was statistically significant for most of the cytokines. Altogether, the removal of dead cells by a density-gradient before *in vitro* culture allows for clearly improved cytokine detection in *i*NKT cells by ICCS.

### Kinetics of *i*NKT cell cytokine production

**Figure 5:**
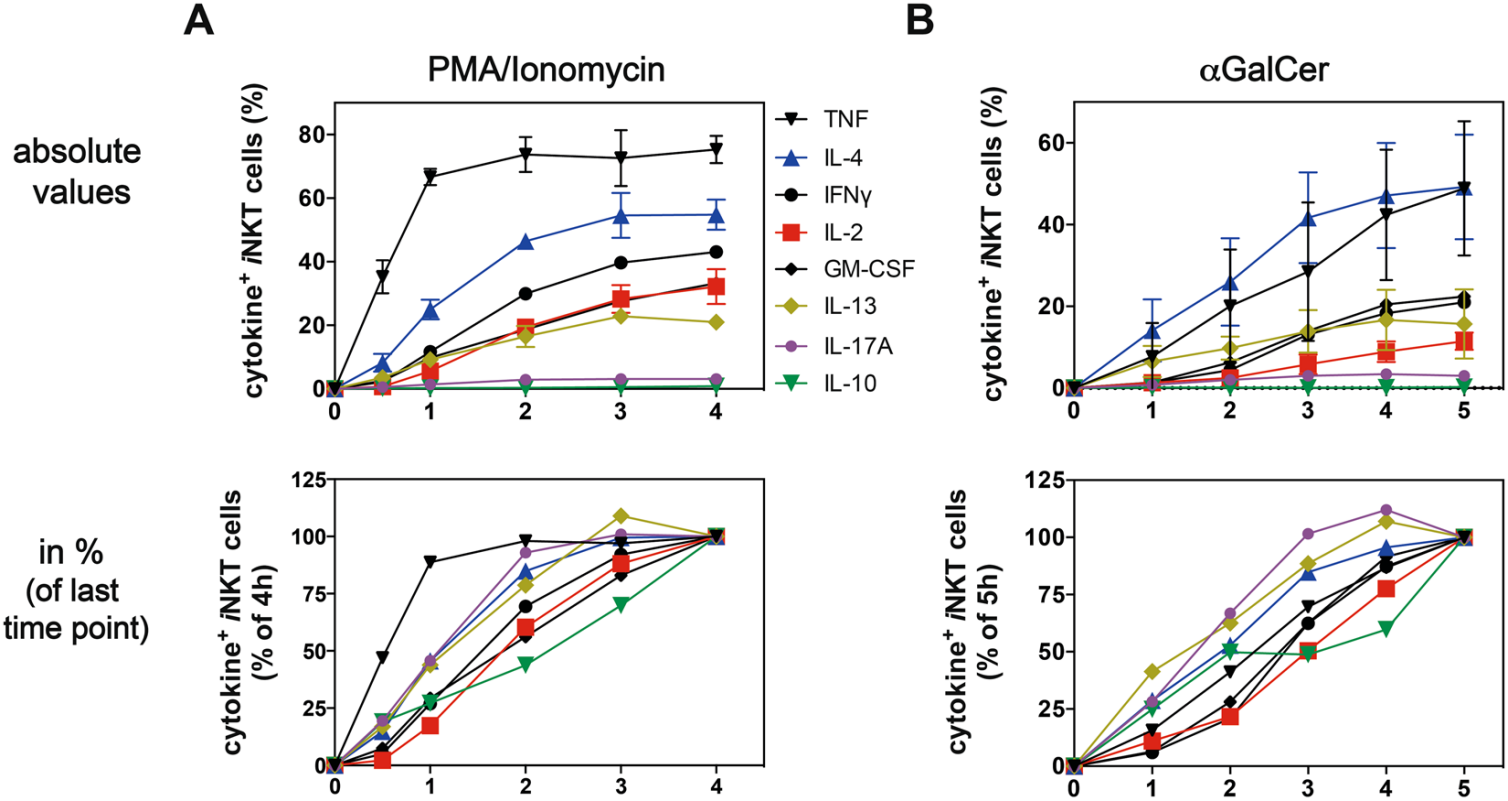
Kinetics of cytokine production by splenic *i*NKT cells. (**A,B**) C57BL/6 splenocytes were purified via a density-gradient and stimulated either for 0.5–4 h with PMA/ionomycin (**A**) or for 1–5 h with αGalCer (**B**) in the presence of the Golgi-transport inhibitors Brefeldin A and monensin. Expression of the indicated cytokines by splenic *i*NKT cells was analyzed by ICCS. A summary graph of the absolute values of cytokine positive *i*NKT cells over time is shown in the top panels. The lower panels show normalized data, with the relative increase of cytokine positive *i*NKT cells at the last time point (PMA/ionomycin = 4 h, αGalCer = 5 h) set to 100%. The graphs summarize the mean values of three independent experiments done in parallel for each stimulation method, with three mice in each experiment.

Having established an optimized protocol for the detection of *i*NKT cell cytokines at the single cell level, we tested its utility by measuring the induction of cytokine production by *i*NKT cells over time. To this end, C57BL/6 splenocytes were stimulated *in vitro* with either PMA/ionomycin or with αGalCer. The cytokines produced by *i*NKT cells were measured between 0.5-4 h after stimulation with PMA/ionomycin or between 1–5 h after stimulation with αGalCer. GM-CSF, IFNγ, IL-2, IL-4, IL-10, IL-13, IL-17A and TNF were measured in parallel by ICCS. Following stimulation with PMA/ionomycin, the percentage of *i*NKT cells producing any of the cytokines measured reached at least 50% of the maximal response after 2 h, with IL-10 constituting the exception requiring 3 h (Fig. 5). Although most splenic *i*NKT cells in C57BL/6 mice have been reported to be NKT1 cells^9^, and the highest frequency of the PMA/ionomycin stimulated cells produced TNF, a high percentage of the cells also produced IL-4, while relatively few cells were positive for IL-2 or IL-13. Therefore, a rapid, multi-cytokine response was elicited by the strong stimulation achieved by PMA/ionomycin.

As expected, the stimulation with αGalCer showed a slightly delayed response. Cytokine production reached more than 50% of the maximal response after 3 h, rather than 2 h. A large proportion of the cells produced IL-4, even larger than the percentage that produced TNF, after antigen stimulation, with a reduced percentage producing IFNγ (Fig. 5). Furthermore, the standard deviation of the cytokine values following αGalCer stimulation tended to be larger than after PMA/ionomycin stimulation. For both methods of stimulating *i*NKT cells, the IL-10 response included the fewest cells, and it also was the slowest to rise.

### Strain comparison of cytokine production

**Figure 6:**
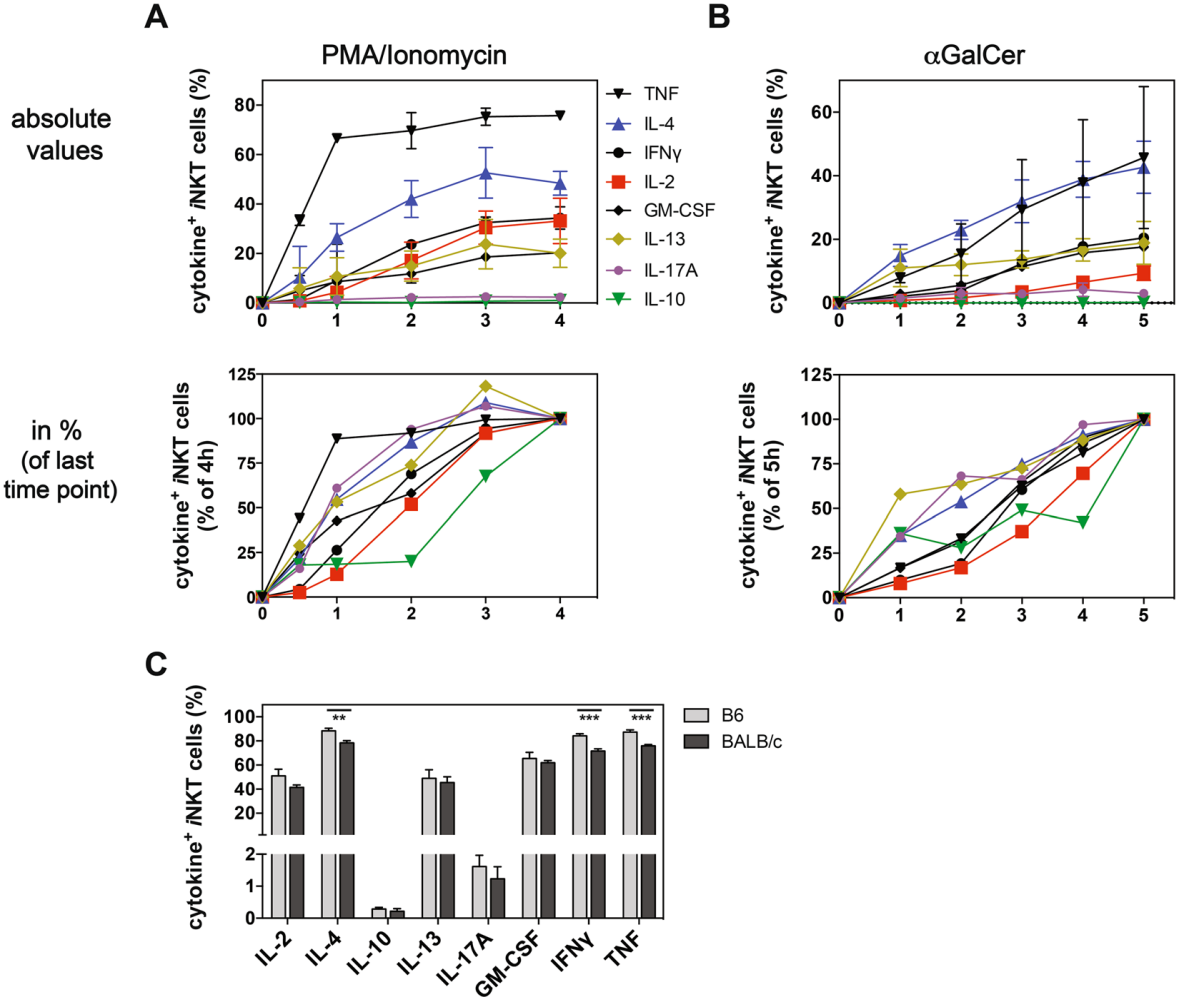
Strain comparison of *i*NKT cell cytokine synthesis. (**A**,**B**) BALB/c splenocytes were purified via a density-gradient and stimulated either for 0.5–4 h with PMA/ionomycin (**A**) or for 1–5 h with αGalCer (**B**) in the presence of the Golgi-transport inhibitors Brefeldin A and monensin. The expression of indicated cytokines by splenic *i*NKT cells was analyzed by ICCS. A summary graph of the absolute values of cytokine positive *i*NKT cells over time is shown in the top panels. The lower panels show normalized data with the relative increase of cytokine positive *i*NKT cells, with the last time point (PMA/ionomycin = 4 h, αGalCer = 5 h) set to 100%. The graphs summarize the mean values of three independent experiments done in parallel for the PMA/ionomycin and αGalCer stimulation, with three mice in each experiment. (**C**) C57BL/6 and BALB/c animals were injected i.v. with 1μg αGalCer and 90 min later splenocytes were purification via a density-gradient and incubated for 2 h *in vitro* in the presence of Golgi-transport inhibitors (Brefeldin A and monensin). The expression of indicated cytokines by *i*NKT cells was analyzed by ICCS. Representative data from one of two independent experiments are shown.

Immune responses in BALB/c mice generally are more biased to Th2 than in C57BL/6 mice^27^. In agreement with this, it has been reported that more Th2-like NKT2 cells are present in the thymus of BALB/c than in C57BL/6 mice^9^. We compared *i*NKT cell production of GM-CSF, IFNγ, TNF, IL-2, IL-4, IL-10, IL-13 and IL-17A in these two strains. C57BL/6 or BALB/c splenocytes were stimulated *in vitro* with either PMA/ionomycin or with αGalCer and the cytokines produced by *i*NKT cells were measured after 0.5–4 h for PMA/ionomycin or 1–5 h for αGalCer. Interestingly, the cytokine response was not significantly different for *i*NKT cells derived from C57BL/6 (Fig. 5) or BALB/c (Fig. 6A,B) splenocytes, irrespective of the *in vitro* stimulation method. To verify that this comparable response was not the result of the *in vitro* conditions, we stimulated C57BL/6 and BALB/c mice *in vivo* with αGalCer for 90 min and measured the *i*NKT cell cytokine response by ICCS. Under these conditions the cytokine response of BALB/c derived *i*NKT cells tended to be lower for all tested cytokines than those of C57BL/6 derived *i*NKT cells (Fig. 6C). However, this difference was small and reached statistical significance only for IL-4, IFNγ and TNF. Together, these data suggest that the cytokine response of splenic *i*NKT cells is largely comparable in C57BL/6 and BALB/c mice *in vivo* and *in vitro*.

## Discussion

The copious amount of some cytokines, like IFNγ, TNF, IL-4 and IL-13, produced by *i*NKT cells *in vivo* makes it possible to detect and quantify them directly *ex vivo*. However, this common practice is suboptimal for other cytokines like IL-2, IL-10, IL-17A and GM-CSF. We describe here an optimized protocol for the detection of *i*NKT cell cytokines *ex vivo*. Furthermore, we describe an improved protocol for the *in vitro* stimulation that allows a significantly improved detection of *i*NKT cell cytokines. Detailed ‘step-by-step’ procedures for these protocols are provided in the Supplemental information.

We first noted that the temperature of the fixation (4 °C vs. 37 °C) significantly increased the detection of IL-2, IL-4 and IL-13 produced by *i*NKT cells, without negatively affecting the detection of other cytokines or staining for molecules on the cell surface (Fig. 1A). The reason for this difference, however, is not clear yet. Furthermore, similar to conventional T cells, the *in vitro* incubation of *i*NKT cells after *in vivo* stimulation in the presence of Golgi-transport inhibitors significantly improved the detection of the cytokines GM-CSF, IFNγ, IL-2, IL-4, IL-13 and IL-17A (Fig. 2). Interestingly, the purification of splenocytes by a density-gradient was essential for the efficient detection of IL-10^+^ *i*NKT cells (Fig. 3). Furthermore, such purification before the *in vitro* stimulation also significantly improved the detection of other *i*NKT cell cytokines (Fig. 4). The effect of the density-gradient centrifugation is likely due to the removal of dead and apoptotic cells. Thus, our data on the functional impairment of *i*NKT cells during *in vitro* cultures are in line with a report showing that *i*NKT cells are sensitive to cell death induced by NAD released from apoptotic cells^28^.

One surprising result of the data presented is the largely comparable cytokine production of splenic *i*NKT cells derived from C57BL/6 and BALB/c mice *in vivo* and *in vitro* (Figs 5 and 6). Immune responses in the BALB/c mice are generally more biased to Th2 than in C57BL/6 mice^27^. In agreement with this is the finding that in BALB/c mice more Th2-like NKT2 cells are present than in C57BL/6 mice^9^. However, in that study^9^ cytokine data where only reported for the thymus and not for the spleen. Therefore, organ specific differences might account for the strain dependent differences observed previously in the thymus^9^ and by us for the spleen. Additionally, NKT2 cells were reported to be located preferentially in the T cell zones of the white pulp of the spleen^29^, and are therefore less easily activated by antigens injected by the i.v. route^29^. This might explain the lack of a marked difference between C57BL/6 and BALB/c mice we observed *in vivo*, but cannot explain the similar outcome we obtained with *in vitro* stimulated cells. The later finding is surprising as the induction of the transcription factor Nur77, which acts as a faithful marker for TCR-engagement in *i*NKT cells^30^, was reported to be equally induced in splenic NKT1 and NKT2 cells following an *in vitro* stimulation^29^. The reason for this discrepancy is currently not know. Nonetheless, our study suggests that the Th2-bias in the BALB/c mouse does not extend to splenic *i*NKT cells.

In summary, the described protocols allow the improved detection of *i*NKT cell cytokines by ICCS *ex vivo* and *in vitro*. The alterations to the protocol outlined here, were not tested for conventional T cells. However, it is possible that some aspects are transferable to conventional, peptide plus MHC class II-reactive T cells.

## Material and Methods

### Mice

All mice were housed under SPF conditions in the vivarium of the La Jolla Institute for Allergy and Immunology (LJI, La Jolla, USA) or the Izmir Biomedicine and Genome Center (IBG, Izmir, Turkey) in accordance with the respective institutional animal care committee guidelines. C57BL/6 and BALB/c mice were purchased from the Jackson Laboratories (Bar Harbor, ME).

### Ethics statement

All mouse experiments were performed under SPF-conditions with prior approval by the institutional ethic committee (LJI ‘Animal Care and Use Committee’; IBG ‘Ethical Committee on Animal Experimentation’), in accordance with national laws and policies. All the methods were carried out in accordance with the approved guidelines and regulations.

### Reagents, monoclonal antibodies and flow cytometry

α-galactosylceramide (αGalCer) was obtained from Kyowa Hakko Kirin (Tokyo Research Park, Tokyo, Japan) or from Avanti Polar Lipids (Alabaster, AL, USA). Monoclonal antibodies against the following mouse antigens were used in this study: CD3ε (145.2C11, 17A2), CD4 (GK1.5, RM4-5), CD8α (53–6.7, 5H10), CD19 (1D3, 6D5), CD44 (IM7), CD45R/B220 (RA3-6B2), CD69 (H1.2F3), CD122 (TM-beta1), CD127 (A7R34, SB/199), GM-CSF (MP1-22E9), IFNγ (XMG1.2), IL-2 (JES6-5H4), IL-4 (11B11, BVD6-24G2), IL-10 (JES5-16E3), IL-13 (13A), NK1.1 (PK136), TCRβ (H57-597) and TNF (MP6-XT22). Antibodies were purchased from BD Biosciences (San Diego, CA), BioLegend (San Diego, CA), eBioscience (San Diego, CA), Invitrogen/ThermoFisher Scientific (Carlsbad, CA), R&D Systems (Minneapolis, MN) or Santa Cruz (Dallas, TX). Antibodies were biotinylated or conjugated to Pacific Blue, eFluor 450, V450, Brilliant Violet 421, Pacific Orange, V500, Brilliant Violet 570, Quantum Dot 605, Quantum Dot 655, eFluor 650, Brilliant Violet 650, Brilliant Violet 711, Brilliant Violet 785, Brilliant Violet 786, FITC, Alexa Fluor 488, PerCP, PerCP-Cy5.5, PerCP-eFluor 710, PE, PE-TexasRed, PE-CF594, PE-Cy5.5, PE-Cy7, APC, Alexa Fluor 647, eFluor 660, Alexa Fluor 700, APC-Cy7 or APC-eFluor 780. Anti-mouse CD16/32 antibody (2.4G2) used for Fc receptor blocking was purified from hyridoma cells in our laboratory or from Tonbo Biosciences. Unconjugated mouse and rat IgG antibodies were purchase from Jackson ImmunoResearch (West Grove, PA). Dead cells were labelled with Blue, Aqua or Yellow Dead Cell Stain Kit (Invitrogen). Flow cytometry and preparation of fluorochrome-conjugated αGalCer loaded CD1d tetramers were performed as described^31,32^. Graphs derived from digital data are displayed using a ‘bi-exponential display’^33^. Vα14*i* NKT cells were defined throughout as live, CD8α^−^, CD19/CD45R^−^, CD44^+^, TCRβ/CD3ε^+^, CD1d/αGalCer-tetramer^+^ cells.

### *In vivo* challenge and cell preparation

Acute activation *in vivo* was induced by i.v. injection of 1 μg αGalCer followed by analysis 90 min later, unless otherwise indicated. Single-cell suspensions from mouse spleen and thymus were prepared as described^34^. In some experiments, intended for intracellular staining of IL-10, splenocytes were purified by use of Lymphoprep (Axis-Shield, Oslo, Norway; and StemCell Technologies, Vancouver, Canada) density gradient centrifugation and by depletion of B cells with anti-CD45R coated magnetic beads (ThermoFisher Scientific). For *ex vivo* experiments intended for intracellular staining of IL-10 or IL-17A, lymphocytes were cultured 2 h in the presence of GolgiPlug and GolgiStop (BD Biosciences, San Diego, CA) at 37 °C.

### *In vitro* stimulation

Splenocytes were stimulated *in vitro* either with PMA and ionomycin (both Sigma-Aldrich, St. Louis, MO) for 4 h; or with 100ng/ml αGalCer for 5 h at 37 °C in the presence of both Brefeldin A (GolgiPlug) and Monensin (GolgiStop). As GolgiPlug and GolgiStop (both BD Biosciences, San Diego, CA) where used together, half the amount recommended by the manufacturer where used, as suggested previously^26^.

### Statistical analysis

Results are expressed as mean ± standard error of the mean (SEM). Statistical comparisons were drawn using a two-tailed Student t-test (Excel, Microsoft Corporation, Redmond, WA; GraphPad Prism, GraphPad Software, San Diego, CA) for all paired samples or otherwise using an ANOVA test (GraphPad Prism). p-values < 0.05 were considered significant and are indicated with *p < 0.05, **p≤0.01 and ***p≤0.001. Each experiment was repeated at least twice, and background values were subtracted. Graphs were generated with GraphPad Prism (GraphPad Software).

## Electronic supplementary material

Supplementary information
