# Supplementary material for: Improved Detection of Cytokines Produced by Invariant NKT Cells
Source: Sci Rep. 2017 Nov 30;7:16607. doi: 10.1038/s41598-017-16832-1 (PMC5709402; doi:10.1038/s41598-017-16832-1)

Supplementary information

Improved Detection of Cytokines Produced by Invariant NKT Cells

Duygu Sag, Müge Özkan, Mitchell Kronenberg and Gerhard Wingender

Supplementary Figure 1. The method of fixation does not influence the intensity of all cytokine staining in *i*NKT cells:

(A and B) C57BL/6 animals were either mock treated or injected i.v. with 1μg αGalCer and 90min later the expression of the cytokines IL-4 (clone 11B11) (A), GM-CSF (MP1-22E9) (B), IFNγ (XMG1.2) (B) and TNF (MP6-XT3) (B) by splenic *i*NKT cells was analyzed. Cells were fixed with Cytofix/Cytoperm for 10 minutes at either 4°C or 37°C as indicated. A summary graph (left panel) or representative data (right panel) are shown. The fluorochromes conjugated to the antibodies utilized are indicated below the histograms. ns = not statistically significant. Representative data from one of at least three independent experiments are shown.

Supplementary Figure 2. The method of fixation does not influence the detection of surface expression

C57BL/6 splenocytes were stained and fixed with Cytofix/Cytoperm for 10 minutes at either 4°C or 37°C as indicated. Cells were electronically gated for *i*NKT cells (live $CD3\varepsilon^+$ $CD8\alpha^-$ $CD19/CD45R^-$ $CD44^+$ CD1d/αGalCer-tetramer$^+$ cells), $CD8^+$ T cells (live $CD3\varepsilon^+$ $CD4^-$ $CD8\alpha^+$ $CD19/CD45R^-$ cells), or B cells (live $CD3\varepsilon^-$ $CD4^-$ $CD8\alpha^-$ $CD19/CD45R^+$ cells). Indicated markers expressed on *i*NKT cells (CD3ε, CD4, CD44, CD69, CD122, CD127, NK1.1), $CD8^+$ T cells (CD8α), or B cells (CD19, CD45R) are depicted as indicated. Representative data from one of at least two independent experiments are shown.

Supplementary Figure 3. The removal of dead cells improves detection of *i*NKT cell cytokines *ex vivo* after αGalCer and *in vitro* after PMA/ionomycin stimulation:

(A) C57BL/6 animals were injected i.v. with 1μg αGalCer and 90 min later splenocytes were either directly (*ex vivo*) or after purification via a density-gradient (+Lymphoprep)

incubated for 2h *in vitro* in the presence of Golgi-transport inhibitors (Brefeldin A and monensin). The expression of the indicated cytokines by *i*NKT cells was analyzed by ICCS. **(B)** C57BL/6 splenocytes were either left untreated (*ex vivo*) or purified via a density-gradient (+Lymphoprep) and stimulated *in vitro* for 4h with PMA and ionomycin in the presence of Golgi-transport inhibitors (Brefeldin A and monensin). The expression of indicated cytokines by *i*NKT cells was analyzed by ICCS. Representative data from one of at least three independent experiments are shown.

Supplementary Protocols

PROCEDURE 1: Measuring of *i*NKT cell cytokines after *in vivo* stimulation (page 7)

PROCEDURE 2: Measuring of *i*NKT cell cytokines after *in vitro* stimulation (page 9)

MATERIALS

Reagents and Solutions

- Mice ***CAUTION!*** *Mice should be kept under specific pathogen free conditions and used at 6–16 weeks of age. All experiments should be done in accordance with national laws and policies and with protocols approved by the institutional ethic committee*.
- 2-Mercaptoethanol (Sigma, #M6250-1L) ***CAUTION!*** *2-Mercaptoethanol is known to be toxic. Avoid contact with skin, eyes and mucous membranes.*
- α-Galactosylceramide (αGalCer) (Kyowa Hakko Kirin #KRN7000; or Avanti Polar Lipids, #867000P) ***CAUTION!*** *If αGalCer is difficult to solubilize, heat it in a water bath at 70°C for 1 h. The final concentration of DMSO in the αGalCer solution should not exceed 10% (vol/vol), otherwise it will denature the CD1d protein*.
- Bovine Serum Albumin (BSA, Sigma, # A4737-100G)
- CytoFix/Perm (BD Biosciences, #554722) ***CAUTION!*** *Reagent contains formaldehyde, a chemical which is harmful if inhaled, in contact with skin or swallowed*.
- Fetal Bovine Serum (FBS, ThermoFisher Scientific, #16000044), heat inactivated
- GolgiPlug (BD Biosciences, #555029) ***CAUTION!*** *Reagent contains Brefeldin A, a chemical which is known to be toxic. Avoid contact with skin, eyes and mucous membranes*.

- GolgiStop (BD Biosciences, #554724) ***CAUTION!*** *Reagent contains Monensin, a chemical which is known to be toxic. Avoid contact with skin, eyes and mucous membranes*.
- Ionomycin (Sigma, #I9657-1MG)
- L-Glutamine (ThermoFisher Scientific, #25030024)
- Lymphoprep (Stem Cell Technologies, #07801)
- Penicillin-Streptomycin (10.000U/mL) (ThermoFisher Scientific, #15140122)
- Perm/Wash buffer (BD Biosciences, #554723)
- Phorbol 12-myristate 13-acetate (PMA, Sigma, #P8139-1MG)
- RPMI 1640 w/o L-Glutamine (ThermoFisher Scientific, #42401018)
- Sodium Azide (NaN_3, Sigma, #S2002-500G) ***CAUTION!*** *NaN_3 is known to be toxic. Avoid contact with skin, eyes and mucous membranes*.
- αCD16/32-Ab (clone 2.4G2) (Tonbo Biosciences, #70-0161-M001)
- αCD45R-microbeads; Dynabeads Mouse Pan B (B220) (ThermoFisher Scientific, #11441D)
- Unconjugated mouse IgG (Jackson ImmunoResearch, #015-000-003)
- Unconjugated rat IgG (Jackson ImmunoResearch, #012-000-003)

<u>Equipment</u>

- Forceps
- Scissors
- Insulin needle (BD Biosciences, #324911)
- 3 mL syringe (BD Biosciences, #309657)

• 70 µm cell strainers (BD Falcon, #08-771-2)

• 50 mL Polypropylene Falcon Tube (Corning Costar, #352070)

• 5 mL round bottom FACS tube (Corning Costar, #352052)

• Flat-bottom 24-well plate (Corning Costar, #3524)

• V-bottom 96-well plate (Corning Costar, #3357)

• Adhesive sealing film (Thermo Fisher, #15036)

Reagent Setup

• BACS buffer (wash buffer): PBS, 1% BSA (vol/vol) 0.1% NaN_3 (wt/vol) (stored at RT)

• MasterMix buffer: BACS buffer with 10 µl/mL of αCD16/32-Ab (clone 2.4G2) and unconjugated IgGs (1:1, 10 µg/mL mix of mouse IgG and rat IgG) (stored at 4°C)

• Surface Staining Cocktail (SF): 50 µl/test MasterMix buffer plus the surface antibodies/reagents. ***CRITICAL!*** *The SF staining cocktail should always contain a reagent for live/dead discrimination. Furthermore, it is recommended that the cocktail contains markers that are not expressed on the cells of interest (dump channel): e.g. for iNKT cells αCD8α- and αCD19-Abs.*

• Intracellular Cytokine Cell Staining (ICCS) Cocktail: 50 µl/test Perm/Wash buffer plus 1 µl/test unconjugated mouse IgG and rat IgG plus the intracellular antibodies

• Complete Medium: Supplement RPMI-1640 medium with 5% (vol/vol) heat-inactivated FBS, 100 U/mL penicillin-streptomycin, 0.05% (vol/vol) 2-Mercaptoethanol, 4 µM L-glutamine

• Perm/Wash buffer: dilute 1:10 with dH_2O

PROCEDURE 1: Measuring of *i*NKT cell cytokines after *in vivo* stimulation

(A) *In vivo* activation of *i*NKT cells and sample collection • *TIMING: 2 h*

1. Inject 1 μg αGalCer *i.v.* (retro-orbital) into BALB/c and/or C57BL/6 mice;
2. After 90 min sacrifice the mice and collect the spleen from injected and control mice.

(B) Purification of splenocytes • *TIMING: 1.5 h*

1. Place a 70 μm mesh cell strainer on top of a 50 mL falcon tube;
2. Transfer one spleen onto the cell strainer;
3. Gently mesh the organ through the filter with a 3 mL syringe plunger and wash the filter with PBS;
4. Centrifuge the cells at 400 *g* for 5-10 minutes at 4°C;
5. Discard the supernatant and re-suspend the pellet in 20 mL PBS;
6. Repeat steps 4&5; ***OPTIONAL:*** *To reduce the total cell number, B cell can be depleted from the splenocytes with 50 μl/spleen of αCD45R-microbeads*.
7. Re-suspend the pellet in 2 mL complete medium;
8. Transfer the cells suspension into a 5 mL FACS tube;
9. Underlay the cell suspension with 1.5 mL Lymphoprep through a glass Pasteur pipette reaching the bottom of the FACS tube; ***CRITICAL STEP!*** *The removal of dead cells via this gradient step greatly improves cytokine detection, in particular of IL-10 (see **Figures 3&4**).*
10. Centrifuge the gradient at 500 *g* for 20-30 min at RT without accelerator or breaks;
11. Collect the interphase containing the cells of interest with a 1 mL pipette;
12. Wash the cells twice with PBS;
13. Re-suspend the pellet with 2 mL of complete medium.

(C) Cytokine accumulation *in vitro* • *TIMING: 2.5 h*

1. Transfer the cells into a well of a flat-bottom 24-well plate;
2. Add GolgiPlug (final concentration 0.5 µl/mL) and GolgiStop (final concentration 0.33 µl/mL) to the cells;
3. Incubate cells at 37°C for 2 h; ***CRITICAL STEP!*** *After in vivo stimulation, the detection of particularly IL-10 and IL-17A is improved by this in vitro step to allow for the accumulation of the cytokines intracellular (see **Figure 2**).*
4. Wash the cells twice with PBS and proceed to the cell staining for flow cytometry.

(D) Cell staining for flow cytometry • *TIMING: 2 h*

1. Aliquot the samples into V-bottom 96-well plates;
2. Add SF staining cocktail to the cells, re-suspend and incubate for 10-30 min on ice in the dark;
3. Cover the plate with adhesive sealing film, and wash the cells twice with PBS;
4. Add 30 µl PBS to the cell pellet;
5. Fix cells by adding and immediate re-suspending with 150 µl of CytoFix/Perm buffer;
6. Incubate for 10 min at 37°C in the dark; ***CRITICAL STEP!*** *Unlike the commonly used 4°C incubation, the ICCS staining in particular of IL-4 is improved by the fixation at 37°C (see **Figure 1**).*
7. Cover the plate with adhesive sealing film, and wash the cells twice with Perm/Wash buffer;
8. Add ICCS cocktail, re-suspend cells and incubate for 20-30 min on ice in the dark; ***PAUSE POINT:*** *The ICCS staining can be incubated overnight at 4°C in the dark.*
9. Wash cells with Perm/Wash buffer and re-suspend cells in 150 µl Perm/Wash buffer;

10. Incubate cells for 5 min on ice to allow unbound antibodies to diffuse out of the cells;
11. Wash once with Perm/Wash buffer and once with BACS buffer;
12. Re-suspend cells with BACS buffer and filter the cells (70 μm mesh) before measuring them on the flow cytometer.

PROCEDURE 2: Measuring of *i*NKT cell cytokines after *in vitro* stimulation

(A) Purification of splenocytes and thymocytes • *TIMING: 1.5 h*

1. Collect the spleen and/or the thymus from BALB/c and/or C57BL/6 control mice;
2. Place a 70 μm mesh cell strainer on top of a 50 mL falcon tube;
3. Transfer one organ onto the cell strainer;
4. Gently mesh the organ through the filter with a 3 mL syringe plunger and wash the filter with PBS;
5. Centrifuge the cells at 400 *g* for 5-10 minutes at 4°C;
6. Discard the supernatant and re-suspend the pellet in 20 mL PBS;
7. Repeat steps 5&6; ***OPTIONAL:*** *To reduce the total cell number, B cell can be depleted from the splenocytes with 50 μl/spleen of αCD45R-microbeads*.
8. Re-suspend the pellet in 2 mL complete medium;
9. Transfer the cells suspension into a 5 mL FACS tube;
10. Underlay the cell suspension with 1.5 mL Lymphoprep through a glass Pasteur pipette reaching the bottom of the FACS tube; ***CRITICAL STEP!*** *The removal of dead cells via this gradient step greatly improves cytokine detection, in particular of IL-10 (see* ***Figures 3&4****).*
11. Centrifuge the gradient at 500 *g* for 20-30 min at RT without accelerator or breaks;
12. Collect the interphase containing the cells of interest with a 1 mL pipette;

13. Wash the cells twice with PBS;
14. Re-suspend the pellet with 2 mL of complete medium.

(B) *In vitro* stimulation of cell suspensions • *TIMING: 2.5 h*

1. Transfer the cells into a well of a flat-bottom 24-well plate;
2. Stimulate the cells with one of following conditions in the presence of GolgiPlug (final concentration 0.5 μl/mL) and GolgiStop (final concentration 0.33 μl/mL):
   (a) PMA (50 ng/mL) and ionomycin (500 ng/mL);
   (b) αGalCer (100 ng/mL);
3. Incubate cells at 37°C for:
   (a) 4 h; **CRITICAL STEP!** 4 hours are required for optimal IL-10 detection (see ***Figures 5&6****);*
   (b) 5 h; ***CRITICIAL STEP!*** *5 hours are required for optimal IL-10 detection as the kinetic with αGalCer is slower than with PMA/ionomycin (see* ***Figures 5&6****);*
4. Wash the cells twice with PBS and proceed to the cell staining for flow cytometry.

(C) Cell staining for flow cytometry • *TIMING: 2 h*

13. Aliquot the samples into V-bottom 96-well plates;
14. Add SF staining cocktail to the cells, re-suspend and incubate for 10-30 min on ice in the dark;
15. Cover the plate with adhesive sealing film, and wash the cells twice with PBS;
16. Add 30 μl PBS to the cell pellet;
17. Fix cells by adding and immediate re-suspending with 150 μl of CytoFix/Perm buffer;

18. Incubate for 10 min at 37°C in the dark; ***CRITICAL STEP!*** *Unlike the commonly used 4°C incubation, the ICCS staining in particular of IL-4 is improved by the fixation at 37°C (see* ***Figure 1****).*

19. Cover the plate with adhesive sealing film, and wash the cells twice with Perm/Wash buffer;

20. Add ICCS cocktail, re-suspend cells and incubate for 20-30 min on ice in the dark; ***PAUSE POINT:*** *The ICCS staining can be incubated overnight at 4°C in the dark.*

21. Wash cells with Perm/Wash buffer and re-suspend cells in 150 μl Perm/Wash buffer;

22. Incubate cells for 5 min on ice to allow unbound antibodies to diffuse out of the cells;

23. Wash once with Perm/Wash buffer and once with BACS buffer;

24. Re-suspend cells with BACS buffer and filter the cells (70 μm mesh) before measuring them on the flow cytometer.

Supplementary figure 1. Sag et al.

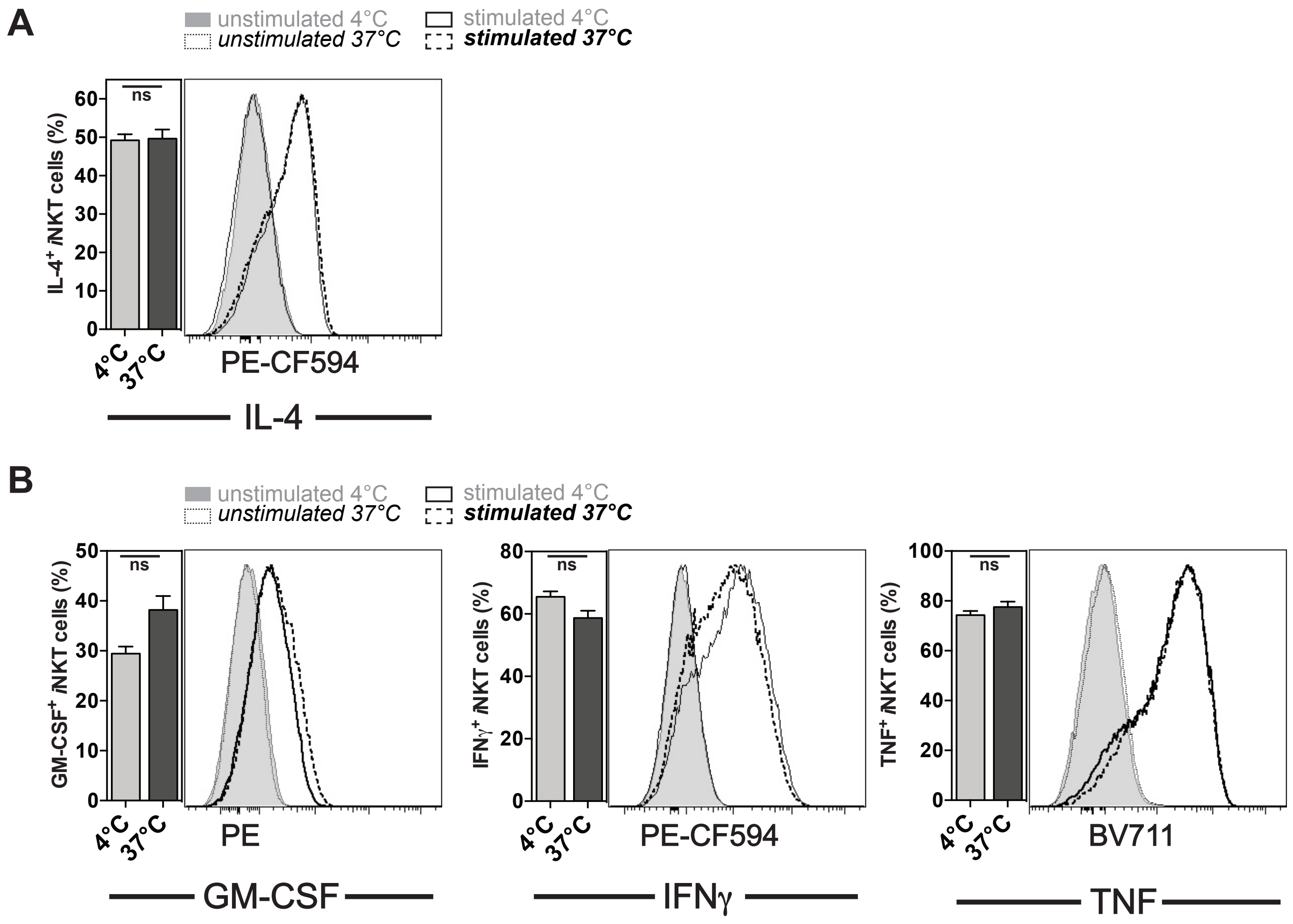

Supplementary figure 2. Sag et al.

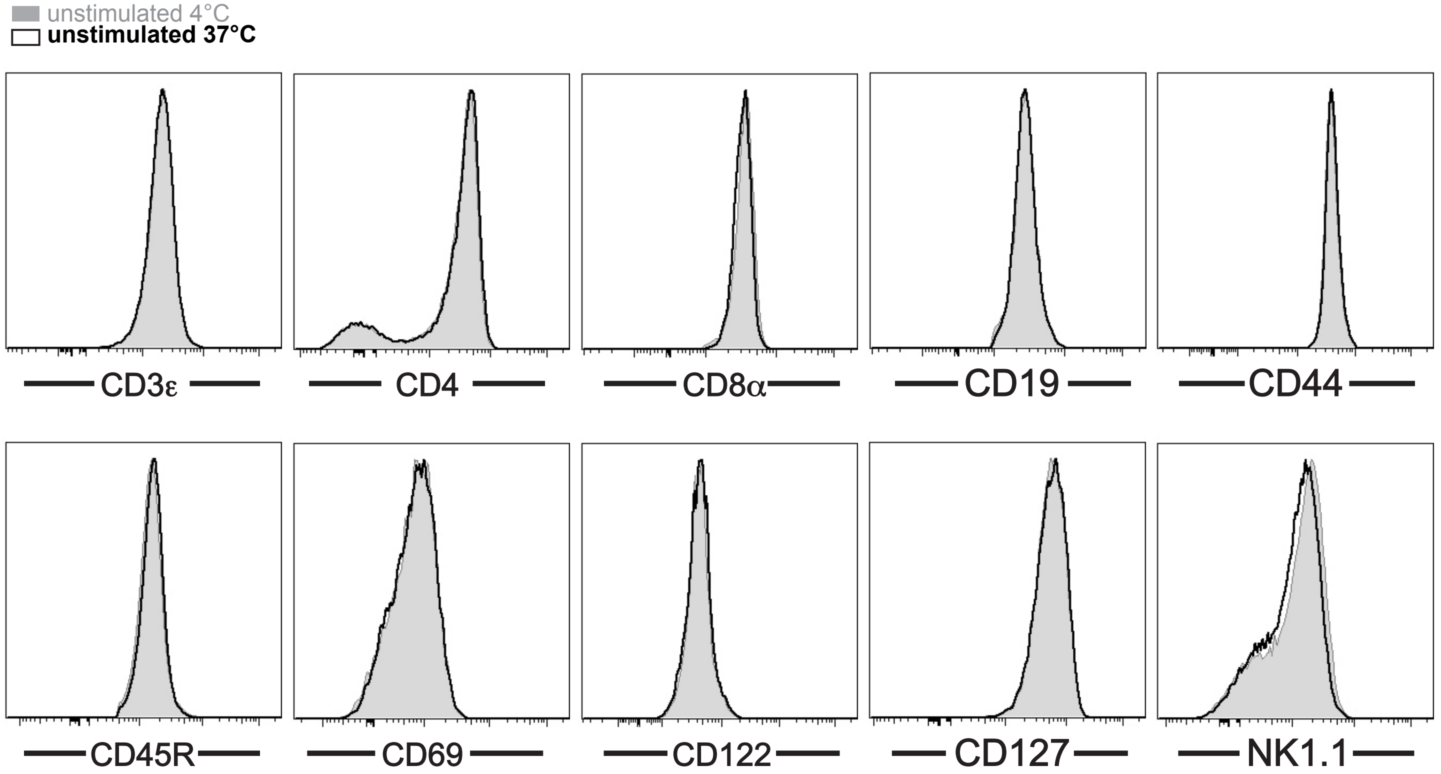

Supplementary figure 3. Sag et al.

A

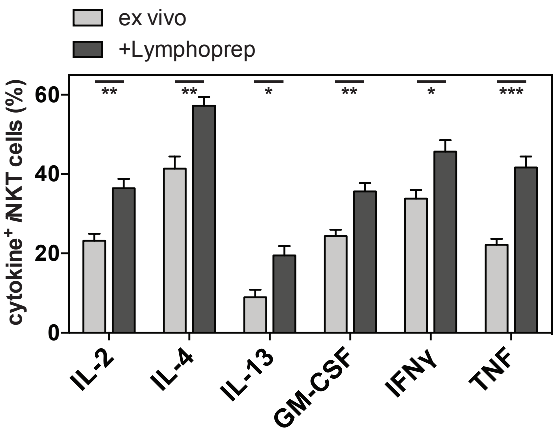

B

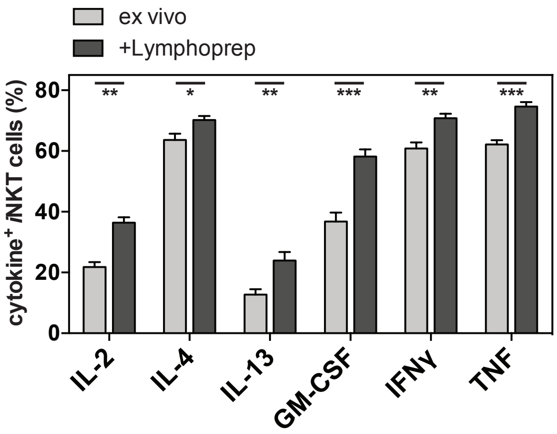

Supplement: Supplementary file 1 — Supplementary information [file 41598_2017_16832_MOESM1_ESM.pdf]
